# A Rosetta stone for epidemiology: genomic risk profile scores contain clues related to modifiable risk factors

**DOI:** 10.1017/S2045796014000651

**Published:** 2014-10-23

**Authors:** John J. McGrath

**Affiliations:** 1 The University of Queensland, Queensland Brain Institute, St. Lucia, Australia; 2 Queensland Centre for Mental Health Research, The Park Centre for Mental Health, Richlands, Australia

In this volume, we are proud to present two invited editorials that provide guidance for future gene-by-environment interaction (G × E) studies. Ruud van Winkel (2014) argues that the field needs to undergo a conceptual shift related to our expectations – we need to shift focus away from the assumption that G × E studies are new tools to help ‘hunt for genes’. Instead, these studies can help partition subgroups of affected individuals, who are more likely to share aetiology and pathogenetic mechansims. This partitioning, in turn, may help to identify biological mechanisms associated with relevant environmental exposures (henceforth exposures) and/or risk alleles. Van Winkel reminds us that clinical diagnostic boundaries (which have been used to define caseness in genomewide association studies; GWAS), do not capture genetic nor neurobiological reality. Nor should they define tight linkages between exposures and psychiatric disorders.

In the second invited editorial, Anna Vinkhuyzen and Naomi Wray outline two modern analytic strategies that can help advance the G × E field (Vinkhuyzen & Wray, 2014). Wray and her colleagues have made key contributions to the field of statistical genetics and were pioneers in the application of Genomic Risk Profile Scores (GRPS) in human genetics (Purcell *et al.* 2009; Wray & Visscher, 2010; Lee *et al.* 2013). Vinkhuyzen and Wray highlight the properties of the GRPS and provide guidance on how this measure can be incorporated into standard epidemiological analytic models. GRPS are quantitative estimates of an individual's aggregated genetic risk for a particular disease. The ability to include a continuous measure of genetic liability in statistical models is an important advance for the field (more on this topic below). Wray and colleagues have also pioneered single nucleotide polymorphism (SNP)-based heritability estimates for quantitative traits (Yang *et al.* 2010, 2013) and disease traits (Lee *et al.* 2011). In their editorial, Vinkhuyzen and Wray outline mixed linear model methods that can incorporate both SNP and exposure data into the matrix of pairwise comparisons.

The G × E field has moved slowly, partly due to the lack of informative datasets (McGrath *et al.* 2013), but also because the traditional methods may have operated as ‘intellectual flypaper’. The field has become somewhat preoccupied with G × E technical issues related to (a) scale (e.g., disease *v.* liability), (b) the nature of the interplay (e.g., dissecting out product–term interaction and gene–environment correlation, etc.) and (c) interpreting the findings (e.g., the biological meaning of results that suggest additive *v.* multiplicative interplay). These issues can be difficult to follow (Zammit *et al.* 2010) which in turn may have stifled scientific progress (thus ‘sticky’). The two editorials provide welcome suggestions on how the field can move forward.

The GRPS offers other properties of interest to the G × E field. As the discovery samples used to generate disease-specific GRPS increase, the scores become more powerful with respect to both disease-linked risk alleles *and disease-linked exposures*. If an exposure is linked to a disease, and if this exposure is influenced by common variants, then exposure-linked variants will be automatically incorporated into the disease-specific GRPS. The task for the research community will be to develop tools to decipher this cryptic information. This property is a ‘by-catch’ for the field – while looking for genes that directly impact on disease risk (i.e., the main goal of the exercise), the GRPS will blindly incorporate clues related to environmental risk factors for the disease of interest. While epidemiologists did not expect this innovation, statistical geneticists may have provided our field with a ‘Rosetta Stone’ to help translate clues from genetics into candidate environmental risk factors.

Significant associations between (a) exposure-linked SNPs *v.* (b) diseases linked to the exposure of interest, are well described in the current literature. For example, the links between risk alleles near certain nicotinic receptors (the CHRNA5-A3-B4 gene cluster) on Chromosome 15 provide an interesting pointer to what might lie ahead for GRPS (Ware *et al.* 2012). A set of SNPs in this region is linked to disease phenotypes such as peripheral arterial disease, chronic obstructive pulmonary disease, airflow obstruction, and lung cancer. In addition, the same SNPs are strongly associated with smoking behaviour and nicotine dependence. Because the link between smoking behaviour and lung disease is beyond reasonable doubt, this pattern of finding strongly suggests that common variants in this region influence smoking behaviour *directly*, and then lung disease *indirectly* via exposure to smoking. It can be deduced that (a) if smoking is a risk-modifying factor for lung cancer and (b) if the GRPS discovery sample includes a reasonable proportion of individuals who smoke, and (c) if smoking is influenced by common variants, then (d) the lung cancer-specific GRPS will be automatically enriched with these smoking-related variants.

**Fig. 1.**
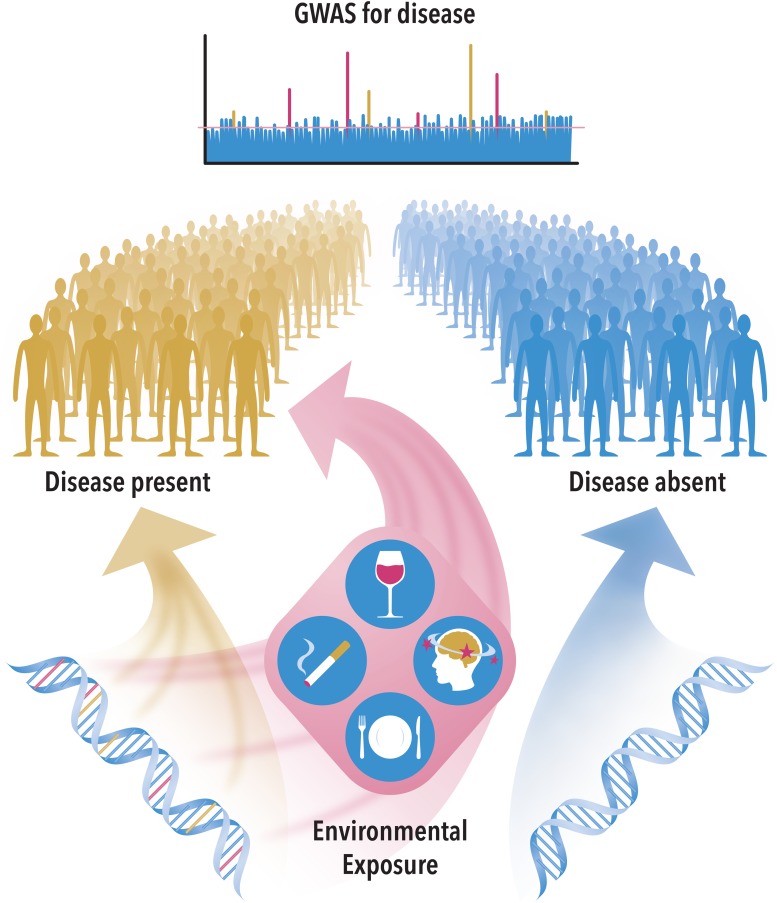
While simplified, the figure outlines how common variants can directly impact on the risk of developing a disease (yellow arrow). In addition, environmental exposures (e.g. diet, substance use, trauma) may also directly impact on the risk of developing this same disease (pink arrow). However, if common variants are associated with the environmental exposure of interest, these will also be identified in the GWAS. GRPS are based on large GWAS studies, and will incorporate information related to direct risk alleles (yellow bars in the GWAS ‘Manhattan’ plot) and exposure related risk alleles (pink bars in the GWAS ‘Manhattan’ plot).

It is a small step to speculate how a well-powered GRPS might amplify the properties of (a) SNPs linked to *one causal variant* that influences *one disease-related exposure v.* (b) SNPs linked to *all causal variants* that influence *all disease-related exposures*. In a thought experiment where we have a well-powered lung cancer-specific GRPS, it would not be necessary to specify the particular risk alleles linked to smoking behaviour. The GRPS, by definition, collects information across the genome, in a hypothesis-free fashion. Thus, the GRPS will blindly harvest SNPs linked to all disease-related exposures – both known and unknown (see Fig. 1).

Armed with a powerful GRPS, the researcher can then go to an independent sample (e.g., a general population sample) – there is no need to enrich the sample for lung cancer or smoking above that found in the general community. This sample needs both SNP data and exposure data related to a *panel of candidate environmental risk factors* associated with lung cancer. This panel will likely contain a mix of some true risk modifiers (i.e., causal agents), some proxy risk indicators that are strongly correlated with risk modifiers, and some candidates that are false leads. First, the lung cancer-specific GRPS needs to be calculated for the independent samples, based on the weighted risk alleles. Everyone will get a score that estimates the liability to lung cancer based on common variants. Next the association between the lung cancer-specific GRPS *v.* each of the candidate exposures included in the panel is explored. It is predicted that causal exposures are more likely to be significantly associated with the GRPS compared with the false leads included in the exposure panel. Proxy risk factors (strongly correlated with causal exposures) will also be more likely to be significantly associated with the GRPS (akin to SNPs in linkage disequilibrium).

This strategy could allow the diligent researcher to ‘fine map’ the environmental exposures of interest in future studies. For example, within a general population sample, a GWAS for the candidate exposure could be done only in those with high disease-specific GRPS (e.g., the top quartile). When the genetic architecture of both the disease and exposure of interest are highly polygenic, the particular risk alleles contributing to a disease-specific GRPS may not be shared by others in the same GRPS strata (i.e., many different risk alleles may push individuals into top GRPS quartile). Van Winkel (2014) reminds us to expect this type of heterogeneity (the ‘unique disease principle’). A GWAS for the candidate exposure in subgroups stratified by disease-linked GRPS may reveal strongly associated SNPs linked to biological pathway of interest to both (a) the disease underpinning the original GRPS and (b) the exposure driving the GWAS. These candidate SNPs can then be taken back to case–control studies for hypothesis-driven G × E analyses. Pathway-specific GRPS can also be derived. For example, nested within the disease-specific GRPS, a subscore could be generated for SNPs in or close to genes involved in biological pathways of interest. As outlined by Vinkhuyzen & Wray (2014), these bespoke GRPS can be included in epidemiological statistical models. The methods could also isolate subgroups that share aetiology or pathogenesis, as proposed by van Winkel (2014). This analytic framework can help reduce the risk architecture ‘search space’, and rank-known candidate exposures and risk alleles. This, in turn, can help catalyse the generation of new hypotheses.

There are important caveats to this application of out-of-sample risk-profiling. Variants may have pleiotropic properties. Thus, a set of SNPs may impact on more than one disease phenotype (e.g., both risk of lung cancer and also the risk of nicotine dependence). Biological plausibility can help weigh up this issue. Environmental exposures often cosegregate in a socially patterned matrix (e.g., education, socioeconomic status, risk of mental illness, risk of substance use, exposure to trauma etc.), thus unmeasured residual confounding may underlie an association between some candidate risk factors and a disease. This confounding may be mirrored in the analytic strategies proposed in the two editorials and in the methods described above.

Twin and family studies have demonstrated that many environmental events (e.g., trauma exposure) are heritable (Kendler & Baker, 2007). Thus, if environmental exposures impact on the risk of adverse mental health outcomes, they may be detected using GRPS in an out-of-sample profile framework. Will these new additions to the G × E toolkit unmask previously unsuspected exposures and risk alleles? This remains to be seen, but it is heartening to know that if we can generate large genotyped sample with detailed information on exposures, we have some innovative methods to explore. The vision outlined by van Winkel (2014) and by Vinkhuyzen & Wray (2014) offers important new leads to guide the research community.
